# Recurrent mutation in *CDMP1* in a family with Grebe chondrodysplasia: broadening the phenotypic manifestation of syndrome in Pakistani population

**DOI:** 10.12669/pjms.316.8115

**Published:** 2015

**Authors:** Sara Mumtaz, Hafiza Fizzah Riaz, Mohammad Touseef, Sulman Basit, Muhammad Faiyaz Ul Haque, Sajid Malik

**Affiliations:** 1 Sara Mumtaz, Human Genetics Program, Department of Animal Sciences, Faculty of Biological Sciences, Quaid-i-Azam University, 45320 Islamabad, Pakistan; 2 Hafiza Fizzah Riaz, Human Genetics Program, Department of Animal Sciences, Faculty of Biological Sciences, Quaid-i-Azam University, 45320 Islamabad, Pakistan; 3 Mohammad Touseef, Human Genetics Program, Department of Animal Sciences, Faculty of Biological Sciences, Quaid-i-Azam University, 45320 Islamabad, Pakistan; 4 Sulman Basit, Center for Genetics and Inherited Diseases, Taibah University Almadinah Almunawwarah, Kingdom of Saudi Arabia; 5 Muhammad Faiyaz Ul Haque, Molecular Genetic Pathology Unit, Department of Pathology & Laboratory Medicine, College of Medicine, King Saud University, Riyadh, Kingdom of Saudi Arabia; 6 Sajid Malik, Human Genetics Program, Department of Animal Sciences, Faculty of Biological Sciences, Quaid-i-Azam University, 45320 Islamabad, Pakistan

**Keywords:** Acromesomelic dysplasia, Dwarfism, *CDMP1*, GDF5, Grebe syndrome, Pakistani subject

## Abstract

Grebe syndrome (OMIM-200700) is a very rare type of acromesomelic dysplasia with autosomal recessive inheritance. We studied a Pakistani family with two affected individuals having typical features of Grebe chondrodysplasia. Patients were observed with short and deformed limbs having a proximo-distal gradient of severity. Hind-limbs were more severely affected than fore-limbs. Digits on autopods were very short and nonfunctional. Index subject also had nearsightedness. However, symptoms in the craniofacial and axial skeleton were minimal. Genetic analysis revealed four base pair insertion mutation (c.1114insGAGT) in gene coding cartilage-derived morphogenetic protein-1 (*CDMP1*). This mutation was predicted to cause premature stop codon. The clinical presentation in this study broadens the range of phenotypes associated with CDMP1 mutation in Pakistani population.

## INTRODUCTION

Grebe syndrome (OMIM-200700), is a very rare autosomal recessive skeletal dysmorphism which exhibits itself as disproportionate dwarfism with profoundly shortened and deformed limbs, but with relatively normal axial and craniofacial skeleton.^1^,^2^ The severity of shortening of limbs progresses in a proximal-distal gradient, with the hands and feet being most affected. The fingers and toes appear bead-like attached with the reduced autopod through delicate bridges. Heterozygous mutation carriers may have an average stature with mild skeletal abnormalities including brachydactyly. Grebe syndrome has been shown to be caused by mutations in *CDMP1* gene at chromosome 20q11.2.^3^-^5^ To date only four families with this condition have been reported from Pakistan. We present another family that showed typical symptoms of Grebe-type chondrodysplasia which segregated with a mutation in *CDMP1*.

## CASE REPORT

**Fig.1:**
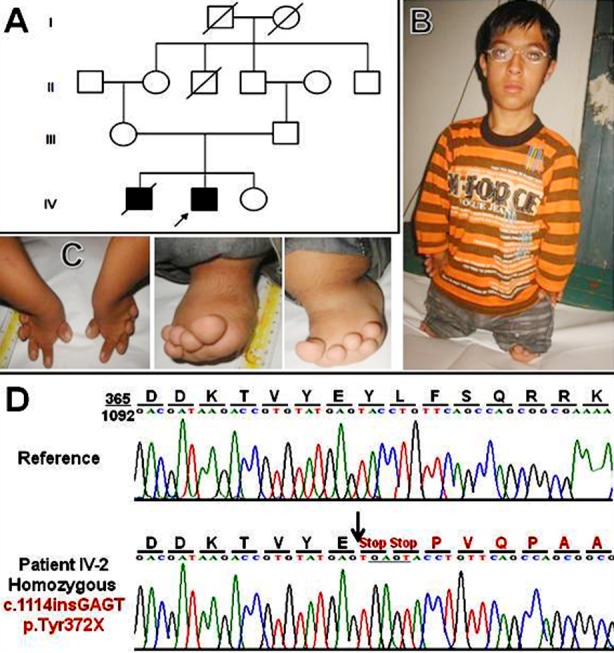
A. Pedigree of the family. Open symbols represent unaffected subjects and filled symbols affected persons. Index subject IV-2 is shown with an arrow below the symbol. B-C. Phenotypic appearance of the subject and his limbs. D. Electrophoretogram showing the reference sequence with amino-acid read in the upper panel while the lower panel depicts 4bp insertion in the second exon of CDMP1 in the index subject.

The family presented here belonged to a rural area of upper Punjab, had a Punjabi ethnicity and an extended household. A pedigree comprising four generations was constructed and two affected male sibs appeared in the fourth generation (Fig.1A). The affected subjects were the product of a consanguineous union (inbreeding coefficient *F*=0.0625). There was no history of maternal drug intake or any other hereditary anomaly in the family. This study was approved by the Ethical Review Committee of the Quaid-i-Azam University, Islamabad.

The index subject (IV-2) was 15 years at the time of examination. He had normal IQ, no formal schooling and was engaged in manual jobs. Clinical assessment showed characteristic symptoms including severe acromesomelic dwarfism affecting all limbs (Fig. 1B,C). The limbs were short and stubby with reduced segments, i.e., stylopods, zeugopods and autopods. The legs were much reduced than arms. Hands and feet harbored short knob like digits, present at the dorsal aspect of the outer rim of autopods. These nubbins had small nails and appeared nonfunctional. The trunk was mildly short and barrel shaped. The craniofacial appearance was unremarkable. However, he had nearsightedness. The subject weighted 35kg. Anthropometric evaluations revealed that he had a standing height of 76cm, sitting height 60cm, arm-span 84cm, head circumference 50cm, neck circumference 28cm, chest circumference 70cm, arm length 36cm, leg length 20cm, and feet were 11cm. Reportedly, the deceased affected subject (IV-1) also had similar phenotypic presentation. His parents were physically asymptomatic.

Owing to the remarkable similarities of phenotype in our subject with the Grebe syndrome, the index subject and his unaffected father were screened for the presence of any pathogenic mutation in the entire coding portion and intron-exon junctions of the *CDMP1* gene. Primer designing, PCR and electrophoresis conditions, and sequence reactions were essentially the same as described earlier.^6^ Sequence reads were generated using ABI 3500 genetic analyzer (Life Technologies, CA, USA) and aligned with the reference sequence from Ensembl Genome Browser using BioEdit software. Sequence analyses of the coding region of *CDMP1* gene led to the detection of a four base-pair insertion mutation (c.1114insGAGT) that results in an immediate stop codon (p.Tyr372X) (Fig. 1D). This mutation is predicted to code a short form of CDMP1 protein consisting of 371 amino acids, without an active domain, which is expected to abrogate the signaling pathways of CDMP1 target cells.^6^

## DISCUSSION

To date, four Pakistani families have been reported with Grebe syndrome while three different mutations were found. The first family was studied by Faiyaz-Ul Haque et al.^4^ with a total 13 effected individuals having an insertion mutation (c.297insC) in *CDMP1*. Basit et al.^6^ investigated another Pakistan family with Grebe syndrome and identified a four base insertion mutation (c.1114insGAGT) in the *CDMP1* gene. Jalil et al.^7^ reported a kindred with Grebe syndrome which showed additional symptom of congenital heart disease. However, molecular analyses of that family were not reported. A fourth Pakistani family was described with a single base pair substitution (c.527T>C) in the same gene.^5^

We present here another family that showed features of Grebe syndrome. The mutation identified in our family is the same as reported by Basit et al.^6^ Although, the family investigated by Basit et al.^6^ originated from Punjab province of Pakistan as well, however, certain differences were evident in the clinical presentation of both families (Table-I). In the family described by Basit et al.^6^, there were six nubbin like digits in the hands; and only one and two toes-like-remnants were observed in the right and left foot, respectively. However, in the present case, both hands and feet had five nubbin like digits. The index subject in the present kindred also had nearsightedness which is a novel association and was not witnessed in the family reported by Basit et al.^6^

**Table-I:** Comparison of clinical presentation of family reported by Basit et al. (2008)^6^ and the present family.

Clinical features	Basit et al. 2008	Present family
Relative normal craniofacial skeleton	Yes	Yes
Relative normal axial skelton	Yes	Yes
Upper and lower limbs	Affected	Affected
Proximo-distal gradient of severity	Yes	Yes
Hands	Postaxial polydactyly/extra nubbins	Normal number of fingers
Feet	Complete agenesis of certain toes	Normal number of toes
Nearsightedness	Absent	Present

The phenotypic differences might be due to variable expressivity of the gene(s) that cause difference in the molecular gradient that is important in the development of fingers and toes during embryonic stages. Nonetheless, it is quite likely that some ancestral mutation is segregating in both families.^8^ In conclusion, the present study gives another evidence of involvement of *CDMP1* in limb morphogenesis and broadens the phenotypes associated with *CDMP1* mutation in Pakistani population.
